# Preparation and Compatibilization of PBS/Whey Protein Isolate Based Blends

**DOI:** 10.3390/molecules25143313

**Published:** 2020-07-21

**Authors:** Maria-Beatrice Coltelli, Laura Aliotta, Vito Gigante, Maria Bellusci, Patrizia Cinelli, Elodie Bugnicourt, Markus Schmid, Andreas Staebler, Andrea Lazzeri

**Affiliations:** 1 Department of Civil and Industrial Engineering, University of Pisa, 56122 Pisa, Italy; laura.aliotta@dici.unipi.it (L.A.); vito.gigante@dici.unipi.it (V.G.); iaia86@live.it (M.B.); patrizia.cinelli@unipi.it (P.C.); andrea.lazzeri@unipi.it (A.L.); 2 Consorzio Interuniversitario Nazionale per la Scienza e Tecnologia dei Materiali (INSTM), 50121 Florence, Italy; 3 IRIS Technology Solutions S.L., Parc Mediterrani de la Technologia, Avda.Carl Friedrich Gauss No. 11, Castelldefels, 08860 Barcelona, Spain; ebugnicourt@iris.cat; 4 Sustainable Packaging Institute SPI, Faculty of Life Sciences, Albstadt-Sigmaringen University, 72488 Sigmalingen, Germany; schmid@hs-albsig.de; 5 Fraunhofer-Institut für Verfahrenstechnik und Verpackung IVV, 85354 Freising, Germany; andreas.staebler@ivv.fraunhofer.de

**Keywords:** biopolyester, poly(butylene succinate), whey protein, compatibilization, polymer blends

## Abstract

In this paper the production of biopolymeric blends of poly(butylene succinate) PBS and plasticized whey protein (PWP), obtained from a natural by-product from cheese manufacturing, has been investigated for the production of films and/or sheets. In order to add the highest possible whey protein content, different formulations (from 30 to 50 wt.%) were studied. It was found that by increasing the amount of PWP added to PBS, the mechanical properties were worsened accordingly. This trend was attributed to the low compatibility between PWP and PBS. Consequently, the effect of the addition of soy lecithin and glycerol monostearate (GMS) as compatibilizers was investigated and compared to the use of whey protein modified with oleate and laurate groups obtained by Schotten-Baumann reaction. Soy lecithin and the Schotten-Baumann modified whey were effective in compatibilizing the PWP/PBS blend. In fact, a significant increase in elastic modulus, tensile strength and elongation at break with respect to the not compatibilized blend was observed and the length of aliphatic chains as well as the degree of modification of the Schotten–Baumann proteins affected the results. Moreover, thanks to DSC investigations, these compatibilizers were also found effective in increasing the PBS crystallinity.

## 1. Introduction

The growing preoccupation about dependence on fossil fuels has led to the development of new materials from renewable resources able to respond to market needs. The push of biobased and biodegradable materials was mainly due to the problem of the disposal of conventional plastics; in fact, about 360 million tons of plastic materials are used everywhere in different sectors and constant growing is expected in the next years [1,2,3]. Important demands are expected for those sectors where biodegradability offers clear advantage for customers and environment, such as single use applications, for which recovery and recycling are important features [4]. The use of biopolymers to replace conventional packaging systems attracts the interest not only of the researchers but also of the industries. Some biopolymers in fact not only come from renewable resources but at the same time several biomaterials can easily compost as an alternative end of life option [5,6,7].

However, some of the renewable biopolymers are plant-based and thus they could compete with food first generation feedstocks [8]. Consequently, the use of a second-generation feedstocks (that means use biomaterials that are byproducts, wastes or residues from the first generation feedstocks), is the preferable option to foreseen [9,10]. At this purpose a valid option can be the production of biopolymeric blends and proteins that are low-cost natural waste. Vegetable as well as animal proteins are natural polymers that, blended with suitable additives, can assume a behavior typical of conventional polymers [5].

Among the available proteins (vegetal proteins like: corn zein, wheat glute soy proteins or animal proteins like: collagen, gelatin and keratin) whey protein is produced in large quantities [11]. In fact, whey is a byproduct of cheese-making and casein of the dairy industry and it constitutes about 20% (*w*/*w*) of the total milk protein [12]. The cheese production increased of 13% in 2017 and 16% in 2018 consequently, the availability of whey protein in recent years has grown even more [13]. Whey is a mixture of globular protein molecules composed by: β-lactoglobulin (β-LG; ~50 wt.%/wt.), α-lactalbumin (α-LA; ~20 wt.%/wt), immunoglobulins (IgG; <10 wt.%/wt), and bovine serum albumin (BSA; <6 wt.%/wt) and other minor protein/peptide components (such as lactoferrin, lactoperoxidase and lysozyme) [14]. The three-dimensional molecular structure as well as the thermal-induced folding/unfolding of β-LG and α-LA have been extensively studied and characterized in literature [15,16,17]. Nowadays the whey protein extracted from dairy industry is valorized for applications in food and pharmaceutical industries [9,18,19]. However, a relevant use will be expected for the production of new polymeric materials to be used primarily in the packaging sector. For example, edible films and coatings for food products have been produced successfully from milk proteins [20]. 

Functional properties, high availability and low market competitive cost of whey proteins make them appropriate to produce transparent, flexible films [21]. Thermoplastics films based on proteins can act as barrier agent towards oxygen and lipids (thanks to the protein structure and to its ability to form cross-linked networks [5]) but, due to their hydrophilic nature they have poor barrier properties to moisture. To improve the barrier properties several cross-linkers have been investigated but their effect on whey proteins is difficult to compare because of the different experimental conditions adopted [21,22,23].

Two techniques have been developed to produce protein-based materials: wet and dry processing [24,25]. In the wet process the protein is dispersed and solubilized with high quantity of a solvent that is then removed by drying. On the other hand, the dry process consists of mixing the proteins with suitable additives (in low moisture conditions) followed by thermo- mechanical shaping (like compression molding, extrusion, or injection molding) [26]. Extrusion and injection molding are widely accepted techniques largely adopted in the plastics industry and for this the acceptance of protein-based bioplastics materials must be studied taking into account the processing techniques adopted from the industries. At this purpose, the blending (dry process) approach is more expedient to scale-up into commercial process by using existing equipment [27].

However, due to the complex macromolecular structure and the large amount of functional groups, proteins can create a series of possible inter-chains reactions that provoke during the extrusion processing an excessive increment of the melt viscosity causing the failure of the process due to the achievement of the maximum torque and pressure values into the extruder. This, combined with the fact that proteins are sensitive to heat, makes very small the operative processing window [28]. During the extrusion process a difficult equilibrium must be reached between the high viscosity that induces mechanical stresses and the protein degradation [29]. The heating process disaggregates and denatures the proteins in the direction of the flow and these changes allow proteins to recombine and cross-link, resulting in an increased glass transition temperature and high melt viscosity [30]. To enlarge the processing window and to facilitate the extrusion process suitable additives such as plasticizers [28] or other thermoplastics polymers [20,31,32] can be added. Indeed, since protein-based films have lower mechanical properties than conventional plastics, the addition of plasticizers and thermoplastics polymer can help to overcome these drawbacks.

Plasticizers increase the free volume and the molecules mobility consequently, as temperature increases above the glass transition, the plasticized protein turns into a soft material easily processable and shaped into desired forms [24,33]. The plasticizer addition not only improves the mechanical properties of films (that are more flexible) but also increases the film permeability [34]. Water is the most effective plasticizer for protein-based films [24]; however, its use is not recommended especially during the extrusion of proteins with bio-polyesters which tend to degrade in the presence of water. Glycerol (C_3_H_8_O_3_) is a low molecular weight, hydrophilic plasticizer that is a good candidate because it has been widely investigated for the thermoplastic processing of proteins [29,35,36,37]. Whey protein isolate sheets plasticized with glycerol and water have been successfully obtained using a co-rotating twin-screw extruder and a slit die [24]. To improve the moisture barrier property, hydrophobic plasticizers derived from citric acid have also been investigated [38]. Their use (in particular, acetyl tributyl citrate (ATBC) and tributyl citrate (TBC)) is interesting because they are mostly used as common nontoxic plasticizers for biopolyesters [39,40,41]. 

Blending proteins with biodegradable polyesters is reported to be a good strategy for improving the processability and mechanical properties of protein-based blends [5]. Researchers have worked on blends of soy protein isolate with fossil based polymers like poly(ethylene-co-vinyl acetate) (EVA) [15,32] but also with biodegradable polymers like poly(butylene succinate) (PBS) [42,43], polycaprolactone (PCL) [44,45], poly(lactic acid) (PLA) and poly (butylene adipate-co-terephthalate)(PBAT) [46]. In this latter case, the aim was mainly preparing compostable materials for packaging.

Among the biodegradable polyesters available on the market, PBS is certainly a promising candidate to be blended with proteins for film production. It is a semi-crystalline polymer synthetized from butanediol and succinic acid [47,48]. The starting mechanical properties of PBS are similar to polyethylene. PBS has excellent biodegradability; good thermal properties and it is very easily processable. In fact, it is suitable for extrusion lamination, blown film extrusion, casting and injection molding. Its good flexibility and its easy film-forming ability make it particularly suitable for film production [40,43,49]. Moreover, it results biodegradable in soil [50]. Despite its good performance, to reduce the costs of the final material, a significant research effort has been made to obtain PBS-based blends and composites containing low-cost ingredients resulting from waste or industry by-products [43]. From this point of view, the study of PBS-based mixtures containing plasticized whey protein (PWP) (available in high quantities) is certainly interesting.

The aim of this work is to investigate PBS-PWP blends with the aim of maximizing the content of PWP (to reduce the costs) and at the same time with an acceptable ductility that allows to use this blend for the production of flexible packaging films and multilayer structures. 

PBS/PWP blends with an increasing amount of PWP (from 30 to 50 wt.%) have been studied in terms of their mechanical properties and melt viscosity observing that the processability could be controlled up to 40% of PWP content and the mechanical properties were worsened due to low compatibility between PWP and PBS. Thus, the PBS/PWP blend containing 40 wt.% of PWP was selected to investigate the effect of several compatibilizers. In particular, fully biobased and biodegradable glycerol monostearate (GMS) and soy lecithin were investigated. To improve further the blend compatibilization also PWP modified with oleate and laurate groups (by Schotten–Baumann reaction [51]) were studied. Four sample where produced: two with oleic chains with a high (OL+) or low (OL−) modification degree and two with lauric chains with a high (LA+) or low (LA−) modification degree. Scanning electron microscope analysis (SEM) allowed to study the blend compatibilization. The results were thus integrated with thermo-mechanical properties investigated by differential scanning calorimetry (DSC) and tensile tests results. 

## 2. Results and Discussion

### 2.1. PBS-PWP Blends First Screening

In the first screening step, various PBS-based blends with different PWP amounts (from 30 to 50 wt.%) were processed and analyzed with the aim of selecting the most suitable PWP percentage that guarantees acceptable melt processability and final thermo-mechanical properties.

From the torque values reported in Table 1 it can be observed that, increasing the PWP content an increment of Torque value is recorded with a maximum of 144 *N*∙cm for PBS/PWP 50/50 blend. This torque increment was expected due to the fact that proteins possess large amount of different functional groups able to induce cross-linking thus resulting in a melt viscosity increment [28]. The high torque value that is obtained with 50 wt.% of PWP can be ascribable to the reaching of a point where the protein phase becomes continuous making the intermolecular crosslinking phenomena more relevant [28].

**Figure 1:**
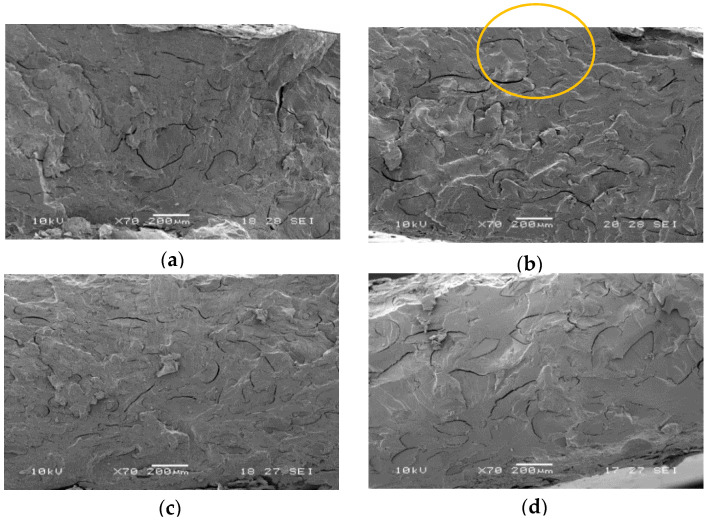
SEM micrographs related to: (**a**) PBS/PWP 50/50; (**b**) PBS/PWP 60/40; (**c**) PBS/PWP 65/35; (**d**) PBS/PWP 70/30. One big domain is circled in (**b**) as example.

From the mechanical properties (also reported in Table 1) it can be observed that the elastic modulus decreases by increasing the PWP content. This effect can be probably ascribed to the glycerol contained into the PWP that partially plasticizes PBS. The elongation at break as well as the stress at break diminish by increasing the PWP content. This decrement can be attributed to the poor stress transfer between the PBS matrix and the protein phase. SEM images (Figure 1) show the protein phase that forms large domains (greater than 100 μm) within the matrix. The shape of PWP domains is not rounded as a result of a not adequately dispersion in the matrix during the extrusion. The incompatibility between PBS and PWP phases is also highlighted by the presence of voids (due to the PWP detachment from the PBS matrix). The size of the PWP dimensions increases with the PWP content and this effect, combined with the poor adhesion, explains the observed decrement of the mechanical properties. In fact, the protein acts as stress intensification factor leading to the marked reduction of the stress at break and the elongation at break. 

From this preliminary screening emerges that the maximum amount of PWP that can be added without incurring in excessive viscosity increment (and thus in processability problems) is 40 wt.% of PWP; in fact, at 50 wt.% of PWP the viscosity brusquely rises up. However, the mechanical properties decrease because of the poor compatibility between the two phases. Therefore, a compatibilization strategy is necessary.

### 2.2. Effect of Compatibilizers on PBS-PWP 60/40 

The blend containing the highest PWP content and acceptable viscosity (PBS/PWP 60/40) was therefore further investigated. In particular, the addition 5 wt.% of soy lecithin (L) and glycerol monostearate (GMS) was studied. Also, a tailored compatibilizer, produced by the modification of the PWP protein by Schotten–Baumann reaction [51], was considered. In fact, in literature, different studies [26,52] have shown that fatty acids are good compatibilizers for mixtures based on plasticized soy protein, whey protein and for collagen-based protein films [53]. In this work the aliphatic chain is linked to the protein, allowing to obtain an emulsifier with an aliphatic long chain and a long protein chain. Therefore, compatibility with the protein should be significantly improved, while the aliphatic segment should be compatible with PBS.

During the first extrusion attempts, 5% of the total PWP content (40 wt.%) the 5 wt.% was modified with four different additives. Blend name and compositions as well as the additives introduced in PWP by Schotten-Baumann reaction are reported in materials and methods—Section 4.

**Figure 2:**
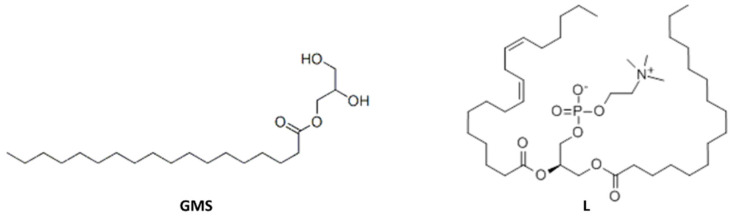
Molecular structure of glyceryl monostearate (GMS) and soy lecithin (L).

It can be observed, from the data reported in Table 2, that the blend PBS/PWP/L containing soy lecithin seems to give improved properties with respect to PBS/PWP 60/40 both in terms of viscosity and mechanical properties. The elongation at break passes from 11.5% to about 30%. On the other hand, the GMS addition causes a torque increment (although this value is still acceptable) if compared to the starting blend PBS/PWP 60/40. This increase could be attributable to the interactions between the additive aliphatic chains (Figure 2), that should place its polar head (represented by the part containing hydroxyls groups), in the protein phase. On the other hand, lecithin is an ionic compound at neutral pH and it is therefore more compatible with the protein phase, rich in polar groups.

**Figure 3:**
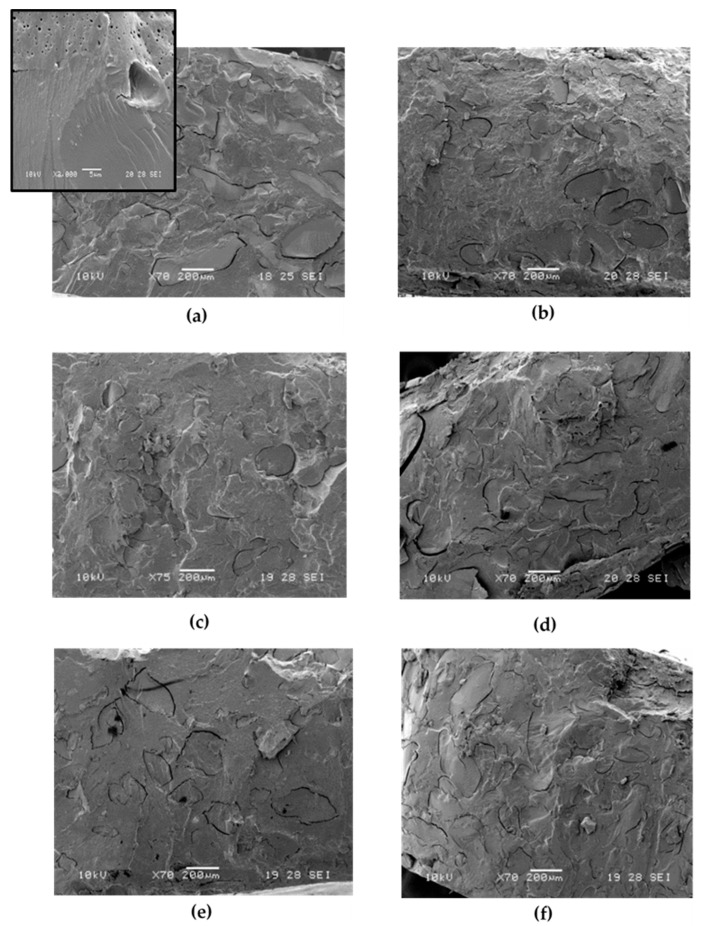
SEM micrographs related to PBS/PWP 60/40 with the following compatibilizers: (**a**) soy lecithin; (**b**) GMS; (**c**) OL+; (**d**) OL−; (**e**) LA+ (**f**) LA−.

From the SEM images (Figure 3) it can be noticed that, compared to the PBS/PWP 60/40 reference blend in which the two phases showed clearly distinguishable cracks at the interface, with the addition of lecithin a better adhesion is achieved. However, the blend containing lecithin, has a very large size of the protein phase that can be attributable to the torque decrement (recorded with this additive) which has disadvantaged the correct mixing of the protein with the polyester phase. Nevertheless, these larger protein phases are quite well attached to the matrix and this is also demonstrated by the improved elongation at break achieved with respect to the reference PBS/PWP 60/40 mixture. On the other hand, GMS seems to not provide a good compatibility with PBS.

**Figure 4:**
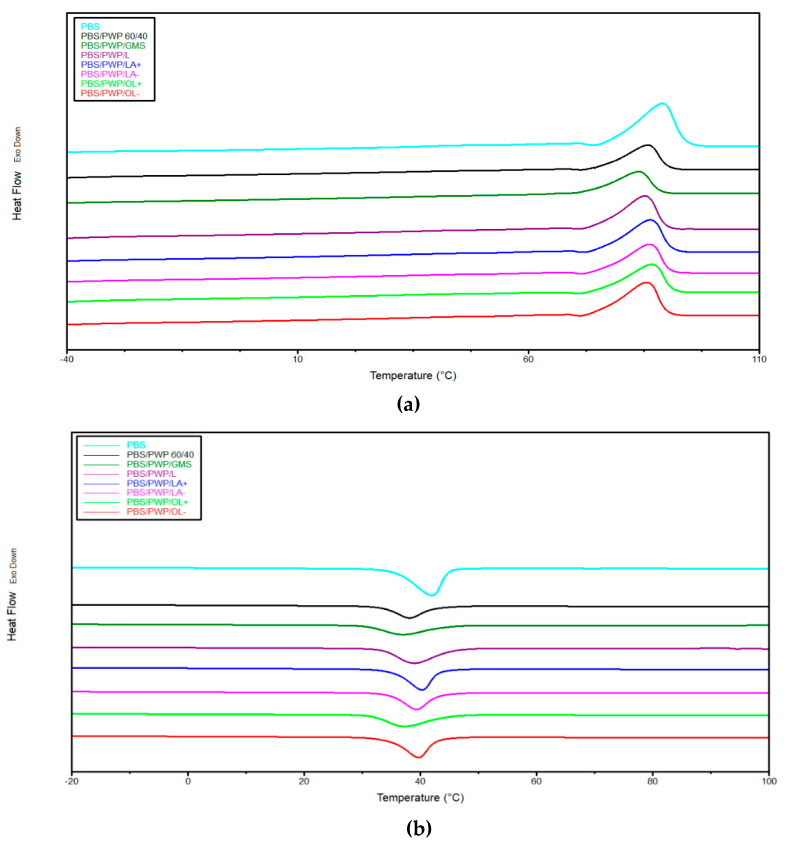
DSC thermograms for: (**a**) second heating and (**b**) cooling scan.

In Table 3 the data of thermal characterization are reported while thermograms are reported in Figure 4. Pure PBS and the reference mixture (PBS/PWP 60/40) were also characterized.

Compared to pure PBS, a decrease in melting temperature and crystallization temperature with the addition of PWP can be observed. The addition of the protein phase (even of large dimensions as seen from the SEM images) slows down the PBS crystallization which passes from 43 to 34% of crystallinity percentage. The use of compatibilizers seems to favor the PBS crystallinity. In fact, a better adhesion allows to the protein phase to act as a heterogeneous nucleation site for PBS. At this purpose, the results of the thermal analysis seem to confirm the poorly compatible effect of GMS which, even from the mechanical results, did not show great improvements compared to the reference mixture. Since GMS does not show any compatibilizing effect, it does not significantly influence the crystallization of PBS that remains almost the same of PBS/PWP 60/40.

PBS/PWP 60/40 mixture was also compatibilized with protein modified by the Schotten–Baumann reaction. Through this reaction, which consists in reacting the protein with the acyl chloride of a fatty acid, hydrophobic chains of oleate and laurate have been linked to the amino acid chains of the plasticized whey protein following the study of Micard et al. [26]. To describe the compatibilization with proteins modified with oleic and lauric group with different degree of modification of the chain, it is necessary to make an organic and complementary discussion by analyzing infrared spectra and then continue with torque, morphology and mechanical tests.

**Figure 5:**
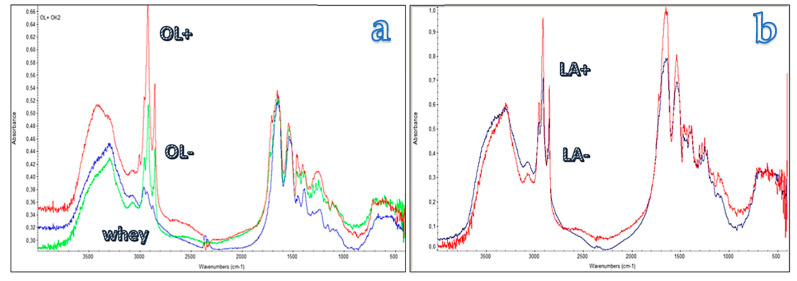
Infrared spectrum of the pure whey protein (green spectrum in (**a**)), and of whey modified with oleic (**a**) and lauric (**b**) groups having different degree of modification.

The infrared spectrum (Figure 5) of the whey protein as it is shows bands at 3300 cm^−1^ attributable to N-H stretching, at 1640 cm^−1^ (amide I) and at 1540 cm^−1^ (amide II) of the bio polyamide whey chains. The modified proteins show an increase in the intensity of the band at 2850 and 2930 cm^−1^ attributable to C-H stretching vibration and thus linked to the presence of the aliphatic chain. The intensity of the band increases by increasing the degree of modification of the protein. 

From the torque values expressed in Table 2 it is possible to note how the length of the chain (C18 and C12) and the different degree of modification affect the viscosity value which increases significantly compared to the PBS/PWP 60/40. It is therefore possible that long aliphatic chains present in a percentage consisting of the mixture interact with each other promoting intermacromolecular interactions. It should be observed that this increment drastically slows down both by decreasing the content of aliphatic chains (OL+ compared with OL−) and decreasing their length. Interestingly an increase in torque was also noticed in the case of GMS addition, having a chain consisting in 18 carbon atoms, thus like the one of OL+. It is evident that a consistent concentration of additives bearing these long aliphatic chains is detrimental for a good processing. However, interestingly the L represents an exception. In fact, despite it has two long fatty acid chains in its molecular structure (Figure 2), it determines a significant decrease in torque. This result is not easy to explain, but it is reasonably related to the capacity of L to interact with the protein polar groups, making them less capable to interact one with the other. Moreover, in these blends the protein phase consists of protein macromolecules surrounded by water and glycerol. The L acts probably inside this phase like a surfactant, contributing to proteins emulsification [54] thus much decreasing the viscosity of this phase. GMS is probably less effective because of its non-ionic feature. The modified protein, on the other hand, can only act as a compatibilizer between PWP and PBS, but not as emulsifier, because the hydrophilic part consists of long proteinic macromolecules contributing in enhancing interactions and reactions with PWP, so they are less effective in decreasing the viscosity of the blend.

The morphological analysis (Figure 3c–f), shows how the use of the modified protein is mirrored into a general good interaction and adhesion between the phases that become wider and very well compacted compared to the reference mixture (PBS/PWP 60/40). In particular, in the mixture with LA− a very good adhesion is showed, but not a good distribution. The phases are not very rounded but tend to lengthen. Instead, in the blend with OL+, a better distribution can be observed with phases that are smaller and rounder. This can explain an increase in torque and, also analyzing the mechanical properties, a considerable increase in elongation at break (a fundamental property for the final application of these mixtures: films and sheets). PBS/PWP/OL−, on the other hand, shows a morphology with wider, elongated domains, evident cracks at the interface which are reflected in a low elongation at break also correlated to a stiffening (in fact the elastic modulus of the blend with OL− is higher).

The use of the protein modified by Schotten–Baumann reaction leads to an increment of PBS crystallization (Table 3). In particular, with LA + and OL− the highest PBS crystallinity content have been recorded. Thus, these results are attributable to the achievement of a better adhesion between the protein phase and the PBS (confirmed also in this case by the mechanical results). The presence of aliphatic groups seems to have a greater nucleating effect on the PBS/PWP 60/40 mixture. Long chains and the presence of a greater quantity of oleic groups favors the formation of aliphatic domains that interact with the PBS. On the contrary, when these long chains are present in low quantities (PBS/PWP/OL−) their nucleating effect is higher. If we consider shorter chains, even if they are present in high quantities (PBS/PWP/LA+), it is instead possible to hypothesize that the nucleating effect is more effective. This suggests an active role in nucleation of the PBS/PWP interface structure.

## 3. Materials and Methods

### 3.1. Materials

**Figure 6:**
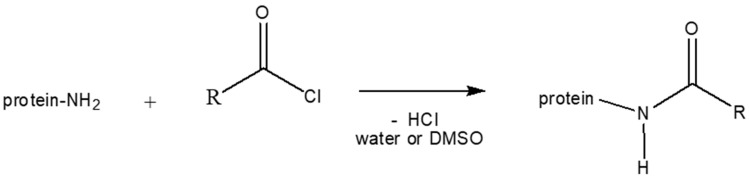
Schematization of Schotten–Baumann reaction.

The list of the materials used in this work is reported below:

- The isolate whey protein (WPI) and the hydrolyzed whey protein (h-WPI) (hydrolysis degree 10%) provided by Davisco Food Int. Inc (Le Sueur, Minnesota, USA).
- Sodium sulphite purchased by Sigma-Aldrich Chemie (Steinheim, Germany).
- Glycerol was provided by Chemsolute Th. Geyer & Co KG (Renningen, Germany).
- The plasticized whey protein (PWP) was prepared by FRAUNHOFER IVV (Freising, Germany). It is constituted by: 43.8 wt.% of isolate whey protein (WPI), 4.9 wt.% of hydrolyzed whey protein (h-WPI), 2.5 wt.% of sodium sulphite and 48.8 wt.% of a Glycerol/ Water mixture [parts ratio 70:30].
- Poly(butylene succinate) (PBS) was purchased from Mitsubishi Chemical Corporation (Tokyo, Japan), trade name GS PLA FD92. It is a copolymer of succinic acid, lactic acid and 1,4-butandiol. It is a semi-crystalline polyester that thanks to its great flexibility can be used for both blown and cast film extrusion.
- The glycerol monostearate (GMS) adopted for this work was purchased from Sigma-Aldrich with trade name of “Glycerol monostearate 40–55” (in which the range of monostearate is 40–55 wt.%).
- Soy lecithin was purchased from Sigma-Aldrich;
- The whey protein modified with oleate and laurate groups (OL+, OL−, LA+ and LA−) with different degree of modification of the chains, were provided by FRAUNHOFER IVV (Freising, Germany). These modified proteins were obtained by the Schotten–Baumann reaction [51]. (Figure 6). The chemical mechanism consists in the reaction of the protein with the acyl chloride of a fatty acid, hydrophobic chains of oleate and laurate are linked to the amino acid chains of the plasticized whey protein.

#### 3.1.1. Blends and Specimen’s Preparation

Blends preparation was carried out on a Thermo Scientific HAAKE Minilab II twin-screw mini-compounder (HAAKE, Vreden, Germany). This equipment is able not only to compound the molten material, but at the same time is able to make torque measurements. Blends name and compositions investigated in this work are reported in Table 4. 

A first screening test was carried out on various PBS blend containing increasing amount of PWP (from 30 up to 50 wt.%). Also, pure PBS was compounded as starting reference. The blend that showed the best compromise between controlled processability, better PWP content (as high as possible) was PBS/PWP 60/40 that contains 40 wt.% of PWP. This formulation was adopted as basis for the successive formulations. In fact, keeping a PLA/PBS ratio of 60/40, the effect of the addition of compatibilizer was investigated. In particular, 5 wt.% of GMS (PBS/PWP/GMS blend name) and Soy lecithin (PLA/PWP/L blend name) were added decreasing the PBS content at 55% wt. 5% wt. of Schotten–Baumann modified proteins were added decreasing the PWP at 35% wt., because they consist mainly of whey, to maintain the content of PWP as close as possible to 40%. The different modified whey proteins (OL+, OL−, LA+, LA−), obtained by Schotten–Baumann reaction, are listed in Table 5 which also contains the protein modification degree.

Before the extrusion, the PBS granules were dried in an oven at 60 °C for 24 h. The mixtures were fed into the Haake mini-extruder by means of a suitable hopper. The processing temperature was set at 130 °C with a residence time of 1 min while the screws rotating speed was 50 rpm. In order to record the torque values, for each extrusion compounding, 6 g of PBS pellets were manually mixed together with the other additives and fed into the mini-extruder. After the introduction of the material, the molten material, pushed by the screws, was flushed in a back-flow channel (with the exit valve of the die closed) for 1 min. During this period, the torque value was recorded as a function of time after 30 s of mixing.

The extruded material was recovered (opening the die valve) after one minute of rotation inside the mini-extruder chamber to ensure a correct mixing. At least five experimental torque measurements were carried out for each blend to assure the reliability and consistency of the test. The final torque value represents the most significant value for the sample as the melt stabilizes. Torque value is capable to give indirect information about the viscosity of the blend during the extrusion.

After the extrusion, the molten materials were transferred through a preheated cylinder, to a Thermo Scientific Haake MiniJet II mini injection molder (HAAKE, Vreden, Germany) for preparation of specimens (Haake bar type 3 with gauge dimensions: 20 × 5 × 1.5 mm) for the successive tests. The cylinder temperature was set equal to the mini-extruder temperature (130 °C) while the injection pressure was set at 500 bar. The molding residence time was 15 s with a mold temperature of 35 °C.

#### 3.1.2. Mechanical Testing

For tensile tests Haake Type 3 dog-bone tensile bars were used. Tensile tests were carried out at room temperature and at a crosshead speed of 10 mm/min on an Instron universal testing machine 5500R equipped with a 10 kN load cell and interfaced with a computer running MERLIN software (INSTRON version 4.42 S/N–014733H). The specimens were tested not before 24 h from their injection molding. At least five specimens were tested for each sample, and the average values reported.

#### 3.1.3. Differential Scanning Calorimetry

In order to investigate the thermal properties of the blends examined, a calorimetric analysis was performed using a Q200-TA Instrument differential scanning calorimeter (DSC, TA Instruments, New Castle, DE, USA) equipped with a RSC cooling system. As purge gas was used with nitrogen set at 50 mL/min. Indium was adopted as a standard for temperature and enthalpy calibration of the instrument. The materials used for DSC analysis have been cut from the HaakeType 3 dog-bone bars. The sampling was carried out exactly in the same region of the injection molded specimens to avoid differences ascribable to different cooling rates in the specimen thickness. Aluminum pans with samples were sealed before measurement, the mass of the samples varied between 10 and 15 mg.

The thermal program was set according to the following procedure: the sample was heated, at 10 °C/min, to 140 °C where was held for 1 min (in order to delete the thermal history of the sample). Subsequently, the sample was cooled to −40 °C at 10 °C/min, maintained at −40 °C for 1 min and then reheated again (at 10 °C/min) up to 140 °C. Melting temperature (*T_m_*) and crystallization temperature (*T_c_*) of the blend were determined by considering the maximum of the melting peak (recorded during the second heating) and at the minimum of the crystallization peak (recorded during the cooling step) respectively. As a consequence, the enthalpies of melting (Δ*H_m_*) and of crystallization (Δ*H_c_*) were determined from the corresponding peak areas in the thermograms.

The crystallinity percentage (*X_c_*) of pure PBS and its blends was determined according Equation (1) [55]:

(1) $$X_{c}=\frac{∆H_{m}}{\frac{∆H_{m}^{{}^{\circ}}}{wt.\%\;of\;PBS}\;}\;$$

where Δ*H°_m_* is the theoretical melting heat of 100% crystalline PBS equal to 110.3 J/g [56].

#### 3.1.4. SEM Analysis

The morphology of the blends was studied by scanning electron microscopy (SEM) using JEOL JSM-5600LV (Tokyo, Japan) and by analyzing the fractured surfaces of the samples obtained by breaking them in liquid nitrogen. Prior to SEM analysis, all the surfaces were sputtered with gold to avoid charge build up.

#### 3.1.5. FT-IR Characterization

Proteins modified by the Schotten–Baumann reaction were also characterized by infrared technique. Infrared spectra were recorded at room temperature in the 500–4000 cm^−1^ range by means of a Nicolet 380 FT-IR (Thermo Fisher Scientific, Madison, WI, USA) spectrometer. Transmission spectra were recorded on KBr disks obtained by grinding with potassium bromide (KBr) powder and pressing with a hydraulic press.

## 4. Conclusions

This work provides a contribution on poly(butylene succinate) (PBS) blends containing plasticized whey proteins isolate (PWP) (a completely renewable and cost competitive resource). In order to obtain a formulation with the highest possible PWP content (thus maximizing PWP valorization and minimizing the material cost), several PBS/PWP blends were prepared and processed by a twin-screw extruder. Since the addition of PWP leads to an increase in the torque (and consequently of the melt viscosity caused by intermacromolecular interactions and cross-linking), it has been seen that the optimal content that allows a controlled processability and the highest PWP content is the compound containing 40 wt.% of PWP.

However, in general the addition of PWP to PBS leads to a decrement of the mechanical properties (lower stress and elongation at break than pure PBS). The loss of these mechanical properties is mainly due to the poor compatibility between the protein phase and the PBS matrix. Consequently, to achieve acceptable mechanical properties, several compatibilizers (soy lecithin and glycerol monostearate (GMS)) were tested. It has been observed that GMS is not a good compatibilizer, while soy lecithin, has a good compatibilizing effect. These results show that the ionic nature of lecithin hydrophilic part is more effective in interacting with protein aminoacidic segments than the hydroxyl groups of GMS. Furthermore, soy lecithin leads to good thermal and mechanical results without the need of an additional chemical modification step. The compatibilizing effect of the PWP itself, partially modified with oleate and laurate groups by Schotten–Baumann reaction, was also investigated. The Schotten–Baumann modified whey samples were then investigated as compatibilizers to enhance the compatibility between the hydrophilic part of the compatibilizer and the whey. As far as concern the modified PWP, all modified proteins guarantee an affordable processability and a higher compatibility (mirrored in the improved mechanical properties). Also, the PBS crystallization degree was modified by the modified PWP proteins that act as nucleating agents for PBS. The best result in compatibilization was achieved by OL− sample that guarantees a high but acceptable torque value and at the same time it provides a good dispersion of the PWP phase. This sample shows also the highest crystallinity content among the Schotten–Baumann modified whey samples. As generally a high crystallization degree is associated with high barrier properties because detrimental for diffusion of gases in the material, this interesting result can be reasonably exploited for preparing multiphase materials with modulated barrier properties through a proper compatibilizer molecular design.

In general, the approach of compatibilization was found useful to improve the processability and properties of PWP/PBS blends. The possibility of modulating the viscosity in the melt as well as the mechanical and thermal properties could allow to extend their use in several application sectors, such as packaging, agriculture or mono-use products, where their biocompatibility and biodegradability could effectively decrease the environmental impact of our human activities.

## Figures and Tables

**Table 1:** Torque values and tensile properties of PBS-PWP first screening blends.

Blend Name	Torque (*N*∙cm)	Elastic Modulus (GPa)	Stress at Break (MPa)	Elongation at Break (%)
PBS	54 ± 1	0.34 ± 0.1	29.2 ± 0.9	227 ± 9
PBS/PWP 70/30	57 ± 1.5	0.25 ± 0.2	16 ± 2	41 ± 4
PBS/PWP 65/35	59 ± 1.5	0.17 ± 0.1	9.6 ± 0.3	13 ± 1
PBS/PWP 60/40	67 ± 1	0.16 ± 0.2	7.8 ± 4.9	11.5 ± 3
PBS/PWP 50/50	144 ± 1.4	0.14 ± 0.2	7.3 ± 0.9	9 ± 2

**Table 2:** Torque values and tensile properties of PBS/PWP 60/40 blend containing different compatibilizers.

Blend Name	Torque (*N*∙cm)	Elastic Modulus (GPa)	Stress at Break (MPa)	Elongation at Break (%)
PBS/PWP 60/40	67 ± 1	0.22 ± 0.02	7.8 ± 2.2	11.5 ± 3.0
PBS/PWP/L	30.7 ± 0.6	0.20 ± 0.03	9.9 ± 1.4	28.9 ± 9.0
PBS/PWP/GMS	91 ± 2	0.23 ± 0.07	7.6 ± 0.8	13.0 ± 2.0
PBS/PWP/OL+	114 ± 2.5	0.24 ± 0.03	11.4 ± 0.7	26.0 ± 4.0
PBS/PWP/OL−	71 ± 1.5	0.37 ± 0.05	10.7 ± 0.4	18.7 ± 3.0
PBS/PWP/LA+	74 ± 1.4	0.20 ± 0.08	10.1 ± 1.0	21.6 ± 2.2
PBS/PWP/LA−	69 ± 2	0.22 ± 0.02	10.7 ± 0.5	20.0 ± 7.0

**Table 3:** Results of differential scanning calorimetry analysis (second heating and cooling).

Blend Name	T_c_ (°C)	ΔH_c_ (J/g)	T_m_ (°C)	ΔH_m_ (J/g)	X_c_ (%)
PBS	41.9	46.2	88.9	47.1	43
PBS/PWP 60/40	38.2	38.5	85.9	37.6	34
PBS/PWP/L	38.9	65.8	85.2	56.9	52
PBS/PWP/GMS	36.9	44.5	83.8	37.8	34
PBS/PWP/OL+	37.2	48.0	86.7	45.2	41
PBS/PWP/OL−	39.7	55.2	85.6	53.7	49
PBS/PWP/LA+	40.3	53.9	86.4	52.7	48
PBS/PWP/LA−	39.3	47.8	86.2	45.3	41

**Table 4:** Blends name and compositions.

Blend Name	PBS wt.%	PWP wt.%	Soy Lecithin (L) wt.%	Glycerol Monostearate (GMS) wt.%	OL+ wt.%	OL− wt.%	LA+ wt.%	LA− wt.%
PBS	100	0	0	0	0	0	0	0
PBS/PWP 70/30	70	30	0	0	0	0	0	0
PBS/PWP 65/35	65	35	0	0	0	0	0	0
PBS/PWP 60/40	60	40	0	0	0	0	0	0
PBS/PWP 50/50	50	50	0	0	0	0	0	0
PBS/PWP/L	55	40	5	0	0	0	0	0
PBS/PWP/GMS	55	40	0	5	0	0	0	0
PBS/PWP/OL+	60	35	0	0	5	0	0	0
PBS/PWP/OL−	60	35	0	0	0	5	0	0
PBS/PWP/LA+	60	35	0	0	0	0	5	0
PBS/PWP/LA−	60	35	0	0	0	0	0	5

**Table 5:** Additives obtained by Schotten–Baumann reaction.

Additive Name	Description	Modification Degree
OL+	whey protein modified with oleic group	1 g oleic acid/g whey protein
OL−	whey protein modified with oleic group	0.3 g oleic acid/g whey protein
LA+	whey protein modified with lauric group	0.5 g lauric acid/g whey protein
LA−	whey protein modified with lauric group	0.25 g lauric acid/g whey protein
